# Rubber Bands as Aids in Stethoscopic Auscultation

**Published:** 1873-09

**Authors:** J. W. Southworth

**Affiliations:** Toledo, Ohio


ART. III.—*Rubber Bands as Aids in Stethoscopic Ausculation.* Notes by J. W. SOUTHWORTH, M. D., Toledo, Ohio.

It was my good fortune a year or so ago, to discover while using RubberBands around the margin of the chest piece of my Stethoscope in order to make it conform better to the inequalities of the surface; that it not only answered the purpose intended, but also fulfilled to my great delight the other most desirable objects, viz: the entire abolition of the roaring tubular quality of the sounds as heard previously, and also rendered them less loud, in fact, *making them correspond almo.t exactly* with those heard by the unaided ear.

But this is not all. By its means I found myself able to auscultate to my entire satisfaction through several thicknesses of clothing, and even through a starched shirt bosom. This latter I have repeatedly tried and always with better success than by the ear alone. Of course the less clothing intervening the better, the skin however need never be made bare.

Fretful children and infants can thus be auscultated without the annoyance and delay, and exposure to cold, incident to removing the chest clothing. The only precautions to be taken are to steady the instrument and press it well against the chest, after smoothing out the folds of the clothing and not letting the latter come in contact with any part but the rubber. It also makes the physical exploration of the "fair sex," whether for thoracic or abdominal affections, a far less indelicate procedure; an advantage which both patient and physician will appreciate.

I hope some of our instrument makers will see this and make the *smaller* chest piece of Camman's instrument with a rim to put a rubber band on.

An ordinary elastic band ½ inch wide by 2 inches in length, found in the stores, will just fit the *larger* chest piece of Camann's stethoscope. It is made to stick on by the aid of a little gum arabic, tragacanth or flour paste, so as to lap over the inner margin of the rim, almost as much as the outer one. The elasticity of the rubber makes it fit snugly, and modifies the vibrations as they are conveyed the rigid tubing from the chest. It is in reality just about the same density as the cartilages of the human ear, thus simulating the normal ear sounds, and doing away with that exaggerated intensity and tubular quality, which, obtains in all the (rigid) tubular stethoscopes, and which misleads most of those who are not experts.

If any are disposed to be sceptical about this let them try it for themselves.

